# Regulation and Function of ILC3s in Pulmonary Infections

**DOI:** 10.3389/fimmu.2021.672523

**Published:** 2021-04-23

**Authors:** Joseph P. Hoffmann, Jay K. Kolls, Janet E. McCombs

**Affiliations:** Center for Translational Research in Infection & Inflammation, Department of Medicine, Tulane University School of Medicine, New Orleans, LA, United States

**Keywords:** ILC3, innate lymphoid cell (ILC), pneumonia, SARS-CoV-2, COVID-19, *Klebsiella pneumoniae*, *Streptococcus pneumoniae*, *Pseudomonas aeruginosa*

## Abstract

Lower respiratory infections are among the leading causes of morbidity and mortality worldwide. These potentially deadly infections are further exacerbated due to the growing incidence of antimicrobial resistance. To combat these infections there is a need to better understand immune mechanisms that promote microbial clearance. This need in the context of lung infections has been further heightened with the emergence of SARS-CoV-2. Group 3 innate lymphoid cells (ILC3s) are a recently discovered tissue resident innate immune cell found at mucosal sites that respond rapidly in the event of an infection. ILC3s have clear roles in regulating mucosal immunity and tissue homeostasis in the intestine, though the immunological functions in lungs remain unclear. It has been demonstrated in both viral and bacterial pneumonia that stimulated ILC3s secrete the cytokines IL-17 and IL-22 to promote both microbial clearance as well as tissue repair. In this review, we will evaluate regulation of ILC3s during inflammation and discuss recent studies that examine ILC3 function in the context of both bacterial and viral pulmonary infections.

## Introduction

Th1 and Th2 helper cell subsets were initially defined by cytokine secretion (1, 2) and this was expanded to other T cell subsets including Th17 cells in the early 2000s, which demonstrated that IL-17 secreting CD4+ T cells arise independent of transcription factors of Th1 (STAT4) and Th2 (STAT6) cells (3–5). However, shortly after the expansion of these Th subsets, it was recognized that many of these cytokines could also be produced by non-T-cell receptor bearing innate cells such as IFNγ-producing NK cells. Th2 cytokines, such as IL-5 and IL-13, were found to be expressed in innate lymphoid cells (ILCs) by several groups (6, 7) and these cells have been subsequently termed group 2 or ILC2 cells. Similarly, cytokines associated with Th17 lineage, IL-17 and IL-22, were originally found in ILCs within tonsils and the gastrointestinal tract (8, 9) and termed group 3 or ILC3 cells. A key early finding showing the functionality of ILC3 cells was demonstrating that they could mediate colitis in mice lacking T cells (9). As opposed to the gastrointestinal tract, where ILC3 cells are abundant, ILC2 cells predominate in the lung, seeding tissues during fetal development (10, 11). However, it has been increasingly recognized that ILC3s play a role in lung immunity. Like other ILC populations, ILC3s require IL-7R signaling and derive from Id2 expressing progenitor cells (12). ILC3s also express the transcription factor RORγt, which differentiates them from ILC1 or ILC2 cells. This review will highlight recent advances in ILC3 biology in the lung.

## Regulation of ILC3 in the Lung

ILC3s localized to the lung are ideally positioned to regulate mucosal immunity within the context of constant exposure to environmental insults. Indeed, in newborn mice, insulin-like growth factor 1 (IGF1)-dependent maturation and expansion of ILC3 precursors in lungs was essential for protection against respiratory pathogens (13). Importantly, disruption of ILC3 development in neonates resulted in increased susceptibility to infection into adulthood, emphasizing the importance of early establishment of these surveyors of lung health. Several chemokines may facilitate ILC3 positioning in the lung. The CXCL13-CXCR5 axis has been implicated in localization of ILC3 to inducible bronchial associated lymphoid tissue (iBALT) structures that develop in the lungs in *Mycobacterium tuberculosis* infection in mice (14), while both CCR6 and CXCR5 were expressed by ILC3s recruited to sites of lung tumors in patients (15). Trafficking of ILC3s to the lung during pneumonia was attributed to CCR4 expression, as deficiency of CCR4 in adoptively transferred ILC3s abrogated homing to the lungs in newborn mice (16). This study also demonstrated the possibility that some lung ILC3 populations may derive from circulating ILC3s. Finally, the CXCR6-CXCL16 axis, which is critical for ILC3 precursor localization to the mouse lamina propria (17), enabled homing of ILC1 and ILC2 cells to the lung under inflammatory conditions (18).

Further studies that define mechanisms for how ILC3s or their progenitors traffic to and function in the lung are much-needed, as research on ILC3s largely centers on the gastrointestinal tract where large populations of these cells reside. This gap in knowledge likely stems from the difficulty of studying these cells, as ILC3s comprise < 5% of total ILCs in the mouse lung (19). Similarly, in human lungs, frequencies of IFNγ+ ILCs were higher than IL-17+ or IL-22+ ILCs, though ILC3 and ILC3-like cells encompassed the highest percentage of total ILCs. However, in chronic obstructive pulmonary disease that is associated with iBALT and chronic infection, the percentage of ILC3 cells were increased compared to healthy lung tissue (20), suggesting that environmental exposures may be key drivers of ILC3 accumulation and that studies in mice will need to include modeling such exposures to study ILC3 biology.

Once in the lung, ILC3s are believed to primarily reside in their resident tissue. Parabiosis studies in uninfected mice revealed that > 95% of all ILCs from various tissues were of host origin (21), though ILC3s were not analyzed in the lungs, perhaps due to the low numbers of these cells at rest (19). However, evidence also supports an increase in circulating ILCs can occur during inflammation. Indeed, helminth infection of mice induced an increase in circulating ILC2s derived from the small intestine and lung (22, 23), though only lung-derived ILC2 were able to migrate back into the lung (23). Commensal bacteria in the intestines of newborn mice were also found to induce expression of CCR4 on ILC3s, enabling subsequent migration to the lungs during pneumonia (16). Nevertheless, analysis of human blood found low numbers of ILC3s in circulation at rest (24), suggesting that while mature ILC3s may migrate during inflammation, the majority of ILC3s may reside and proliferate within their resident tissue.

As ILC3s do not bear T-cell receptors, other factors including cytokines, alarmins, and co-stimulatory molecules can mediate ILC3 stimulation to induce effector function. ILC3s provide immune surveillance of the lung at the steady state, delivering immediate innate protection after host exposure to pathogens. During inflammation, IL-1β and IL-23 stimulate ILC3s to produce IL-17 and IL-22 (8, 25), which in turn regulate epithelial barrier function and mediate host response to infections (8, 26, 27). In the lungs, these cytokines function to enhance production of antimicrobial proteins, facilitate barrier repair through promotion of epithelial cell proliferation, and augment neutrophil recruitment, resulting in increased clearance of pulmonary pathogens (28–30). Thus, activated ILC3s are well-poised to provide immediate and direct action toward foreign antigens. Importantly, ILC2s may also provide IL-17-mediated benefits upon infection. Indeed, ILC2s from nasal polyps of cystic fibrosis patients or skin lesions of psoriasis patients transdifferentiated to an ILC3-like cell—expressing RORγt and producing IL-17—upon ex vivo stimulation with IL-1β, IL-23, and TGFβ (31, 32). Therefore, given the abundance of ILC2 in the lung, their potential for plasticity could contribute to ILC3-attributed functions during inflammation.

**Figure 1:**
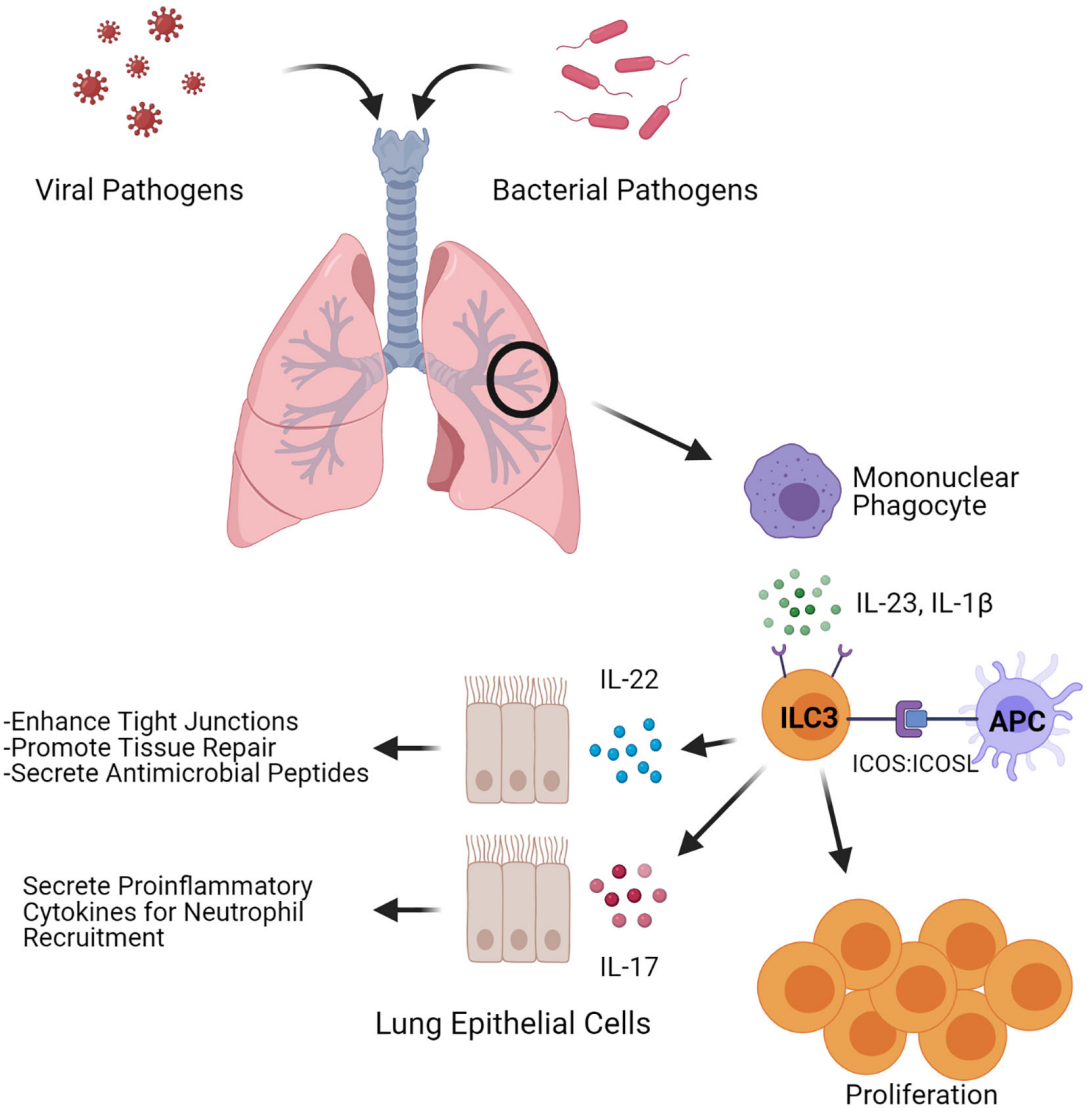
ILC3-induced antimicrobial and tissue regenerative responses. Depiction of factors that influence both inflammatory and regenerative responses in ILC3s upon microbial pulmonary infections. During an infection, ILC3s can be stimulated through ICOS: ICOSL interactions or by the cytokines IL-1β and IL-23. Stimulated ILC3s expand and secrete the cytokines IL-22 and IL-17. Figure created with BioRender.com.

The inducible T cell costimulatory molecule (ICOS) may also stimulate ILC3s in the lung. Differential expression of ICOS, which is also expressed on T cells, has been observed in both mouse and human ILC3s (33, 34). However, the role of this molecule in regulating ILC3 function remains to be fully determined. Mice deficient in ICOS had no change in the total amount of ILC3 in the lung at rest compared to their wild-type counterparts (19), though < 2% of ILCs studied were ILC3s. As not all ILC3s express ICOS, it is possible in the deficient background the balance between ICOS+ and ICOS- ILC3s is shifted, especially since the total population of ILC3s in the lung at rest is very low (19, 20). Indeed, we have shown administration of a neutralizing anti-ICOS antibody to mice prior to bacterial infection resulted in a decrease in *Icos* expression as well as expression of *Il17* and *Il22* after infection with *Klebsiella pneumoniae* (33). In addition, ex vivo stimulation of ILC3s isolated from *K. pneumoniae* infected mice with ICOS ligand (ICOSL) resulted in proliferation of cells (33), indicating the ICOS : ICOSL pathway may be important in mediating ILC3 function and proliferation (**Figure 1**). Interestingly, mouse and human ILC2s express both ICOS and functional ICOSL (35) which can stimulate ICOS+ Treg cells (36), raising the possibility that a coordinated interplay could also exist between ILC3 and ICOSL-expressing ILC2. Such interactions could play a key role in regulating ILC3 activation and providing a swift response upon pathogen presentation in the lungs.

Recent data also suggests a role for the aryl hydrocarbon receptor (AhR) in mediating ILC3 function in the lungs. AhR is an environmental sensor expressed in barrier tissue cells that is critical for ILC3 maintenance and function in the gut (37). Within the environment of the lungs, AhR tunes immune responses to a variety of insults through regulation of ILC3s. Indeed, *Ahr* ^-/-^ mice displayed a decrease in ILC3s during pulmonary paracoccidioidomycosis (38), indicating this receptor could be critical for expansion or recruitment of ILC3 to sites of infection within the lung. In support of this, activation of AhR resulted in recruitment of ILC3 during chronic exposure of mice to ozone (39).

## ILC3s in Bacterial Pneumonia

Even in an era of antimicrobial treatments, pneumonia remains the leading cause of morbidity and mortality in children aged 28 days to 5 years (40, 41). Among the most common etiological bacterial agents in these cases of pneumonia are pathogens such as *Streptococcus pneumoniae, Streptococcus pyogenes, Pseudomonas aeruginosa*, and *Klebsiella pneumoniae* (41–43). Since the discovery of the cytokine IL-17 in 1993 and its receptor in 1995, IL-17 has been demonstrated to be critical in protection against extracellular bacteria and fungi. Further, the coregulated cytokine IL-22 has been shown to promote epithelial integrity and tissue repair at barrier surfaces such as in the gut and lung following inflammation (44). As ILC3s are maintained in lung tissue and can rapidly produce IL-17 and IL-22 upon stimulation, it is clear that they play an important role in the innate immune response against bacterial pneumonia (45).

Though the discovery of ILC3s is recent, there have been numerous studies that have linked ILC3s to bacterial clearance in lung infections. A recent study illustrated that antibody depletion of IL-17 decreases mouse survival against *K. pneumoniae* by 50%. Further, IL-17 induction occurs within the first 3 hours of infection suggesting it is mediated by innate cells such as ILC3s or γδ T cell rather than Th17 cells. In T- and B-cell-deficient *Rag2^-/-^* mice, the dominant source of IL-17 is ILC3 cells. Depletion of these cells using anti-CD90 or *Rag2^-/-^* mice that also are deficient in *Il2rg* (a common cytokine receptor for IL-7, among other cytokines) ablated IL-17 expression and exacerbated pulmonary infection with *K. pneumoniae* (46). It is important to recognize that due to limited ILC3 depletion models, these studies only illustrate that ILC3s are sufficient to clear infection in T-cell-deficient mice. Further studies are required to determine whether ILC3s are required for clearance. Using single cell RNA sequencing, we determined IL-17+, IL-22+, and ICOS+ ILC3s are imperative to protection against carbapenem resistant *K. pneumoniae* in a murine challenge model (33). This study also demonstrated that lung burdens in *Rag2/Il2rg* ^-/-^ mice can be significantly reduced through the addition of exogenous IL-22.

ILC3s also play a role in the clearance of *S. pneumoniae* in murine models of lung infection. One group found that lethal challenge with intranasal *S. pneumoniae* resulted in increased IL-22, IL-17A, and IL-17F expression in lung tissue within 24 hours suggesting a rapid innate response (45). ILC3s appeared to be sufficient for this response as infected *Rag2^-/-^* mice had no change in IL-22 levels compared to wild-type controls, while *Rag2/Il2rg^-/-^* mice were unable to produce IL-22 upon *S. pneumoniae* infection. While these models demonstrate the utility of ILC3s in response to *S. pneumoniae*, future studies are required to demonstrate their necessity. Boosting of ILC3 function could prove therapeutic, as treatment with flagellin at the time of *S. pneumoniae* infection enhanced IL-22 expression in ILC3s and decreased bacterial burdens in mice (45). This was supported by previous findings that treatment with flagellin enhanced ILC3 production of IL-17 and IL-22 in the intestinal lamina propria and spleen (47, 48). The importance of ILC3s in *S. pneumoniae* infections in the neonatal period was demonstrated by Gray et al. This group found that in newborn mouse lungs, 90% of the cells producing IL-22 carried the phenotypic markers of ILC3. Depleting ILC3s by administering diphtheria toxin to *RORγt^iDTR^* newborn mice dramatically increased their susceptibility to *S. pneumoniae* and all mice succumbed to infection within 20 hours. Adoptive transfer of ILC3s into the ILC3-depleted mice restored their resistance to *S. pneumoniae* (16). More recently, it was found that intranasal administration of interleukin 7, an important factor for RORγt+ cell survival and homeostasis, increased the number of RORγt+ innate T cells in the lung, enhanced expression of IL-17A, and reduced bacterial burdens upon *S. pneumoniae* challenge (49).

Many additional studies since the early 2000s characterize the importance of early IL-17 and IL-22 expression in the clearance of bacterial lung infections. Though these studies do not specifically investigate ILC3s, the ability of ILC3s to readily produce IL-17 and IL-22 upon stimulation suggests they are integral in an early IL-17/IL-22 response (44, 50). Additionally, IL-17 evolutionarily predates RAG, the protein needed for adaptive immune responses, as demonstrated by its function in invertebrates (51). This suggests that animals developed IL-17-dependent innate immune responses against bacterial pathogens before the advent of Th17 cells. In one of our previous studies using a murine model of *K. pneumoniae* infection, we found that IL-17R knockout mice had significantly higher bacterial burdens 24 hours post-infection than their wild-type counterparts (52). A similar study infected IL-17R knockout and wild-type mice with *K. pneumoniae* intratracheally and found that 100% of the IL-17R knockout mice succumbed to infection within 48 hours. By comparison, only 20% of the wild-type mice succumbed to infection within the same time frame (53). Interestingly, intratracheal administration of recombinant CXCL5 was able to decrease bacterial burdens in IL-17R knockout mice within 2 hours of treatment (52). Though CXCL5 is predominantly expressed in epithelial cells, ILC3s have been shown to secrete CXCL5 upon stimulation, further implicating them in bacterial immunity. It has also been demonstrated that IL-23-dependent IL-17 production was the most important for survival against *K. pneumoniae* challenge in adult mice (54). This is noteworthy as IL-23 is a potent activator of ILC3s (55). Highlighting the importance of IL-17 and IL-22 in response to *K. pneumoniae* challenge, we demonstrated that bacterial burdens in the lungs dramatically increase upon depletion of both IL-17 and IL-22 (30).

IL-17 has also been demonstrated to play a key role in immunity to *S. pneumoniae*. In a murine model it was demonstrated that systemic depletion of IL-17 at the time of infection resulted in persistent bacterial burdens in the nasopharynx detectable at day 21 post infection (56), suggesting that IL-17 expression at the time of infection may prevent pneumococcal colonization. Another study found that following pneumococcal vaccination, 95% of mice that produced > 0.3 ng/mL IL-17A upon antigen stimulation were protected from bacterial colonization (57). A murine intranasal vaccination study further illustrates the importance of IL-17A in pneumococcal immunity, as IL-17A neutralization abolished all vaccination protection while IFNγ neutralization had no impact on vaccine efficacy (58). Though this is likely indicative of a Th17 response, ILC3s do not produce IFNγ suggesting they are playing a role in the IL-17 dependent protection (12).

Both IL-17 and IL-22 have proven critical in clearing *P. aeruginosa* lung infections. A recent study using human sputum samples found that patients with the highest levels of IL-17 prior to being placed on a mechanical ventilator did not get ventilator-associated pneumonia (59). The importance of IL-17 was supported in a murine lung infection model using *P. aeruginosa*-infused agar beads. In this study, IL-17R knockout mice had inhibited clearance of *P. aeruginosa* infections. Of note, the authors used a clinical isolate from a cystic fibrosis patient and found that IL-17R knockout mice had exacerbated bacterial burdens and increased weight loss 14 days post-infection. Further, in innate immune cells, IL-17 production appeared to be dependent on ILC3s as 90% of the IL-17-producing CD3- cells in the lung carried the phenotypic markers for ILC3s (60). Regarding IL-22, it was demonstrated in mice that absence of IL-22 in *P. aeruginosa* pneumonia enhanced neutrophil recruitment thus exacerbating lung pathology (61). Supporting this, a recent study found that IL-22 upregulated IFNλ expression in a murine *P. aeruginosa* pneumonia model. Increased IFNλ correlated with decreased neutrophil recruitment and knocking out IFNλ led to exacerbated lung inflammation and pathology (62).

## ILC3s in Viral Lung Infections With Implications for COVID-19

Studies investigating ILC3s in viral lung infections are very limited, with much of the focus centered on ILC2s (63). However, there have been numerous studies on the effects of IL-22, a key cytokine of ILC3s, in influenza infections. Though IL-22 does not appear to reduce viral titers, it has been demonstrated to reduce disease severity though its functions in tissue repair and regeneration (64, 65). A recent study by Hebert et al. found that inflammation from influenza infection could be significantly reduced by knocking out IL-22 binding protein (IL-22BP, *Il22ra2*), a soluble inhibitor to IL-22 (66). Additionally, this group showed that *Il22ra2^-/-^* knockout mice had enhanced tight junctions during influenza infection promoting tissue integrity (67). Supporting this was the finding that addition of exogenous IL-22 in a murine model of influenza infection limited tissue damage (68).

Expression of IL-22 and IL-17 during viral lung infections also promotes prevention of secondary bacterial infections. Secondary bacterial infections commonly occur after moderate to severe respiratory viral infections and are a significant contributor to morbidity and mortality (69). During the 2009 H1N1 influenza pandemic, up to 26% of case-patients had a bacterial co-infection, which was associated with longer stays in the ICU and a need for mechanical ventilation (70, 71). Currently it is believed that primary infection with a virus impairs the function of mucus and cilia in clearing otherwise normally nonpathogenic bacteria resulting in opportunistic infection (69). As such, it stands to reason that the regenerative properties of IL-22 secreted by ILC3s may function in prevention of secondary bacterial infections. This was recently demonstrated using a murine model of influenza A (IAV) viral infection followed by a secondary *S. pneumoniae* bacterial infection. The group found that within 2 days of IAV infection there was upregulation of IL-1β, IL-23, and most importantly IL-22. Further, the group found an increase of RORγt cells and IL-22+ ILC3s in the lung. While IL-22-deficient mice had no change in viral clearance, these same mice had dramatically impaired survival after *S. pneumoniae* secondary infection (68). A similar study used transgenic IL-22BP knockout mice infected with influenza followed by *Staphylococcus aureus* or *S. pneumoniae* challenge. The study found that IL-22BP knockout mice had increased bacterial clearance and decreased mortality from secondary bacterial infection, and improved airway epithelial integrity (72).

IL-17 production during influenza infections was also demonstrated to promote the clearance of secondary bacterial infections. One study using a murine model of IAV infection followed by *S. aureus* challenge found that overexpression of IL-23 during infection resulted in enhanced production of IL-17 and IL-22 and promoted bacterial clearance (73). It was subsequently demonstrated that IAV infection prior to secondary *S. aureus* pneumonia inhibited IL-1β production, decreasing IL-22 and IL-17 expression and worsening the *S. aureus* infection. Overexpression of IL-1β during IAV infection rescued the generation of IL-17 and IL-22 and promoted bacterial clearance (74).

The emergence of severe acute respiratory syndrome coronavirus 2 (SARS-CoV-2), which causes the acute respiratory disease COVID-19, has been one of the most severe pandemics and public health crises of the last century. As a newly emerged virus, much remains to be elucidated on effective immune responses distinguishing severe and mild disease. As COVID-19 is a respiratory disease that shares some symptoms with influenza, it is possible that ILC3 production of IL-17 and IL-22 also may serve to limit disease severity. In addition to benefits in influenza infection (65, 72), it was recently demonstrated that IL-22 promotes immunity against respiratory syncytial virus; these benefits of IL-22 may also extend to SARS-CoV-2 infection (75). Similar to influenza, it has also been demonstrated that secondary bacterial infections are common in COVID-19 patients. One study found that of 3,338 total COVID-19 patients, 14.3% developed a secondary bacterial infection (76). It is likely IL-17 may also play a role in preventing secondary bacterial infections in COVID-19 patients, though its role has yet to be demonstrated.

Recently, a function for ILCs has been demonstrated in COVID-19. Evaluation of the blood from COVID-19 patients found that severely infected individuals had fewer ILC1, ILC2, and ILC precursor cells than those with mild disease. Additionally, ILCs in severely infected individuals had higher expression of CD69, a marker for activation and tissue homing. The decrease in blood ILCs coupled with the increase in CD69+ ILCs in severely infected individuals suggests that there is more tissue homing to the lungs in severe infections (77). Silverstein et al. corroborate these findings by establishing that a higher ILC abundance in the blood was associated with less time spent in the hospital. Further, hospitalized individuals with COVID-19 had 1.78-fold fewer ILCs in the blood (78). Taken together, these studies illustrate a correlation between decreased ILCs in the blood periphery and severe SARS-CoV-2 infection. As current data regarding ILCs in COVID-19 infections are obtained though analysis of blood, further studies are required to elucidate their exact role in COVID-19 infection since ILC3s are predominantly tissue resident.

## Discussion

Overall, regulation of ILC3s in both normal and disease states remains an understudied area of research. It is clear these cells contribute significantly to mediating disease as an imbalance of ILC3 has been linked to both COPD and asthma (20, 25). Given their role in barrier protection from invading pathogens, ILC3s or their cytokines could be an ideal target for development of immunotherapies. For example, several groups have FDA approval to study IL-22 in COVID-19 (79, 80) (completed, findings pending). Therefore, it is imperative to develop a complete understanding of how these cells are regulated within the microenvironment of the lungs, which should enable discovery of novel targets for immunotherapeutic development.

## Author Contributions

JH, JK, and JM all contributed to writing and editing of the manuscript. All authors contributed to the article and approved the submitted version.

## Funding

JM is supported in part by U54 GM104940 from the National Institute of General Medical Sciences of the National Institutes of Health, which funds the Louisiana Clinical and Translational Science Center, and R01 AI149119. JK and JH are supported by R35HL139930 and R01 AI149119 from the National Institute of Heart, Lung, and Blood, Diseases and the National Institute of Allergy and Infectious Diseases.

## Conflict of Interest

The authors declare that the research was conducted in the absence of any commercial or financial relationships that could be construed as a potential conflict of interest.
